# Integrating Photon-Based Techniques to Probe Structural and Phonon Dynamics in Bacterial Cellulose

**DOI:** 10.3390/polym17182544

**Published:** 2025-09-20

**Authors:** Levente Csóka, Bunsho Ohtani

**Affiliations:** 1 Faculty of Informatics, ELTE Eötvös Loránd University, 1053 Budapest, Hungary; 2 Nonprofit Organization touche NPO, Sapporo 060-0004, Japan; bunshoohtani@gmail.com

**Keywords:** bacterial cellulose, photon to phonon conversion, X-ray diffraction, nuclear magnetic resonance, photoacoustic spectroscopy, defect states, bandgap engineering, optoelectronic properties, electron traps

## Abstract

Bacterial cellulose, a biopolymer synthesised by microorganisms, exhibits remarkable structural, optical, and electronic properties. This study utilised a range of photon- and electron-based techniques, including X-ray diffraction, proton nuclear magnetic resonance (^1^H-NMR), photoacoustic spectroscopy, and scanning electron microscopy, to thoroughly characterise BC. While XRD and NMR directly employ photons to probe the structure and composition, PAS indirectly converts absorbed photons into phonons to evaluate optoelectronic features. SEM revealed a dense nanofibrillar network with fibrils measuring 10–75 nm in diameter. XRD confirmed the crystalline nature of BC, identifying characteristic peaks associated with cellulose Iα. ^1^H-NMR relaxation analysis differentiated between the ordered and disordered cellulose regions. PAS determined an optical bandgap of 2.97 eV and identified defect states between 3.6 and 2.9 eV, including a prominent peak at 3.35 eV, likely resulting from oxygen vacancies, hydroxyl modifications, or UV-induced rearrangements. These defects modify BC’s electronic structure, suggesting potential for bandgap engineering. The integration of these complementary techniques provides a multidimensional understanding of BC’s morphology, crystallinity, and electronic behaviour, underscoring its potential in bioelectronics, advanced composites, and biomedical applications.

## 1. Introduction

Bacterial cellulose is a unique form of cellulose produced by certain bacteria, particularly those of the genus *Acetobacter* [1,2]. Produced extracellularly, bacteria secrete cellulose fibrils that form a three-dimensional network. Unlike plant-derived cellulose, bacterial cellulose is synthesised as a pure crystalline nanostructure, free from lignin or hemicellulose [3]. Bacterial cellulose primarily consists of cellulose I, with a higher proportion of Iα allomorphs than plant cellulose. The Iα structure has a triclinic configuration and is less stable than the monoclinic Iβ structure. The predominance of Iα contributes to the high crystallinity, mechanical strength, and water-holding capacity of BC. These structural characteristics, along with the absence of lignin and hemicellulose, result in unique properties that render bacterial cellulose valuable for various applications in biomedicine and food technology [4]. Previous studies have demonstrated that bacterial cellulose fibrils possess unique optical properties, making them suitable for diverse applications.

Cellulose inherently functions as an electrical insulator, characterised by flat energy bands that indicate localised charge carriers and necessitate high-energy photons for electronic transitions [5,6]. Intriguingly, our prior research on electromagnetic excitation processes in cellulose demonstrated the existence of electronic vibration bands and excitons, suggesting a more complex interplay of photonic and vibrational behaviours than typically observed in simple dielectric materials [7]. These findings hinted at significant non-radiative energy dissipation pathways, prompting the present investigation into the specific mechanisms of photon-to-phonon conversion within bacterial cellulose fibrils.

These complex photophysical phenomena can be attributed to localised electronic states, defects, or interactions with molecular vibrations; however, they do not imply free electron movement within cellulose molecules. When light interacts with a material surface, it can induce various processes, including the conversion of photons to phonons [8]. The light-induced non-radiative recombination of electron–hole pairs during de-excitation in a sample is a complex phenomenon occurring at atomic and subatomic levels [9]. Upon light absorption, electrons are excited from the valence band to the conduction band, thereby creating electron–hole pairs. Ideally, these pairs recombine radiatively and emit photons. However, non-radiative recombination can occur through various mechanisms, such as Auger recombination or phonon-assisted processes [10]. These non-radiative pathways involve the transfer of energy to lattice vibrations (phonons) or other charge carriers rather than the emission of light. The efficiency of this conversion depends on several factors, including the electronic and vibrational structures of the material, temperature, and the energy of the incident photons.

The phonon generation process begins with the absorption of incident photons by the material, which excites electrons to higher energy states [11]. As these excited electrons relax back to their ground state, they can transfer their energy to the lattice vibrations of the material, thereby generating phonons [12,13]. This process, known as phonon emission, occurs via electron–phonon coupling. The emitted phonons contribute to the thermal energy of the material and propagate through the lattice, thereby affecting its physical properties and thermal conductivity. Understanding this light-to-phonon conversion is crucial for applications in photonics, thermoelectrics, and energy-harvesting devices, where controlling the energy flow between light and matter is essential [14].

Despite its importance, research on the specific mechanisms by which photons are converted into phonons in these fibrils remains limited. This study aims to elucidate these mechanisms in bacterial cellulose fibrils by integrating photon- and electron-based microscopy, spectroscopic and diffraction techniques, considering various influencing factors such as electronic and vibrational structures, and incident photon energy, thereby providing insights into the efficiency and specific processes involved.

## 2. Materials and Methods

### 2.1. Materials

Nata de coco bacterial cellulose was kindly supplied by Thongaumphai’s production, Thailand. It was boiled in deionised water until a neutral pH (approximately 7) was reached. The bacterial cellulose was then purified in a 0.1 M NaOH(Sigma Aldirch, Budapest, Hungary) solution at 80 °C to eliminate non-cellulosic materials. It was subsequently washed until a neutral pH was achieved and then dried in an oven at 105 °C overnight.

### 2.2. Methods

#### 2.2.1. Scanning Electron Microscopy

Scanning electron microscopy images were acquired using a JSM-6390 microscope. Magnification(JEOL, Tokyo, Japan), micrometre markers, and acceleration voltage values are presented in the respective micrographs.

#### 2.2.2. Solid-State Low-Field ^1^H-NMR Measurements

Solid-state, low-field ^1^H-NMR measurements were performed to examine homonuclear dipolar interactions between hydrogen nuclei within bacterial cellulose samples. Samples were prepared by drying at 105 °C for 72 h, then placed into borosilicate glass tubes (0.8–1.0 mm wall thickness, 10 mm diameter, 100 mm length) sealed with rubber caps. Tubes were positioned in a low-field magnetic chamber and allowed to equilibrate for 30 s to align spins. A low magnetic field (B_0_ = 0.5 T) was applied to investigate the interactions. To obtain ^1^H spectra, a direct polarisation experiment was performed, subjecting the ^1^H nuclei to a brief (microsecond) radiofrequency pulse. The resulting free induction decay was recorded in the time domain after a 45° pulse. The acquired FID curves were deconvoluted to resolve individual spectral components and enhance data quality. Subsequent analysis was performed using PeakLab software(Version 1.08.01.) to extract relevant deconvolution information from the FID line shapes in both the time and frequency domains.

#### 2.2.3. Photoacoustic Spectroscopy

A custom-built photoacoustic cell, featuring a stainless-steel body with an internal volume of approximately 0.5 cm^3^, a micro-electromechanical system microphone module (SparkFun MEMS Microphone Breakout, INMP401(InvenSense Inc. San Jose, CA, USA)), and a quartz-glass window (transparent across the 250–1000 nm measurement range), was utilised. The cell was temperature-stabilised at 298.0 K using a block heating-cooling bath to minimise temperature fluctuations during measurements. Bacterial cellulose sample around 8 mm in diameter was placed inside the cell, and PA spectra were recorded at room temperature under a nitrogen atmosphere. Monochromatic light (~0.2 mW/cm^2^) was provided by a Bunkokeiki(Suntek Services, Mumbai, India) monochromator and intensity modulated at 80 Hz. The PAS signal, detected by the MEMS microphone module within the cell, was amplified and analysed using a digital lock-in amplifier. To account for variations in light intensity across different wavelengths, the PAS signal was normalised using carbon black (graphite) powder as a reference [7].

#### 2.2.4. Reversed Double-Beam Photoacoustic Spectroscopy

The photoacoustic spectroscopic technique is centred on acoustic wave generation from absorbed photons. A custom-built photoacoustic cell, featuring a MEMS microphone module and quartz glass window (transparent within 250–1000 nm), was employed to minimise external effects. The RDB-PAS procedure utilised wavelength-scanned monochromatic light (600–250 nm) and a 35 Hz-modulated near-infrared (940 nm) LED. The RDB-PAS signal, indicative of the total trapped electron density, was documented without normalisation. The experimental environment for RDB-PAS consisted of a static methanol-saturated nitrogen atmosphere. Prior to RDB-PAS, nitrogen gas saturated with methanol vapour was passed through the cell for 10 min at 30 mL/min.

The RDB-PAS measurement apparatus involves alternating modulated and continuous-light beams. In this method, continuous wavelength-scanning light excites electrons into electron traps, while simultaneously modulating monochromatic light detects the photoabsorption of electron-filled ETs, recording the photoinduced trap-filling spectrum. Before the measurement, nitrogen gas saturated with methanol vapour was circulated through the cell to irreversibly capture positive holes, thereby preventing the disappearance of trapped electrons through reactions with the positive holes.

#### 2.2.5. X-Ray Diffraction

X-ray diffraction patterns were recorded using a Rigaku SmartLab X-ray (Agilent Technologies, Santa Clara, USA)diffractometer. The diffractometer was operated with Cu-Kα_1,2_ radiation at a wavelength of λ = 0.1540 nm under room temperature settings. A scan speed of 10 deg/min and a scan step of 0.02 deg were used. Background subtraction was performed during the processing of the XRD data.

## 3. Results

### 3.1. SEM Characterisation

**Figure 1:**
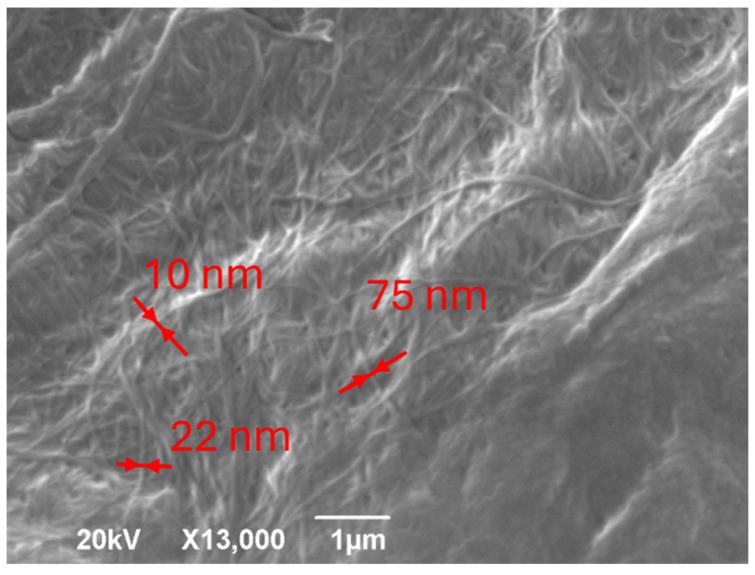
Scanning electron microscopy (SEM) image of bacterial cellulose nanofibrils at 13,000× magnification. The image shows a dense network of intertwined nanofibrils with varying diameters and lengths, demonstrating their high aspect ratios and complex three-dimensional structures.

Scanning electron microscopy analysis of bacterial cellulose revealed its unique nanofibrillar network structure, as depicted in Figure 1. The micrographs show a dense, interconnected web of nanofibrils with diameters ranging from 10 to 75 nm as indicated by red arrows. These fibrils appear as long, thin strands that intricately intertwine to form a highly porous matrix. This characteristic structure significantly contributes to the high surface area and water-holding capacity of bacterial cellulose [15]. Furthermore, the SEM images highlight the remarkable uniformity of the fibrils and the conspicuous absence of other cellular components, which are distinctive features of bacterially synthesised cellulose compared with its plant-derived counterpart. The high magnification and resolution afforded by SEM enabled detailed observation of the fibril arrangement, specifically revealing the presence of ribbon-like structures and extensive interconnections between individual fibrils. These detailed images provide invaluable insights into the morphology and structural properties of bacterial cellulose, which are crucial for understanding its behaviour in various applications. The direct use of electron probing in SEM was instrumental in generating these high-magnification images, thereby revealing the intricate nanoscale features of the bacterial cellulose samples.

### 3.2. Solid-State Low-Field ^1^H-NMR Characterisation

**Figure 2:**
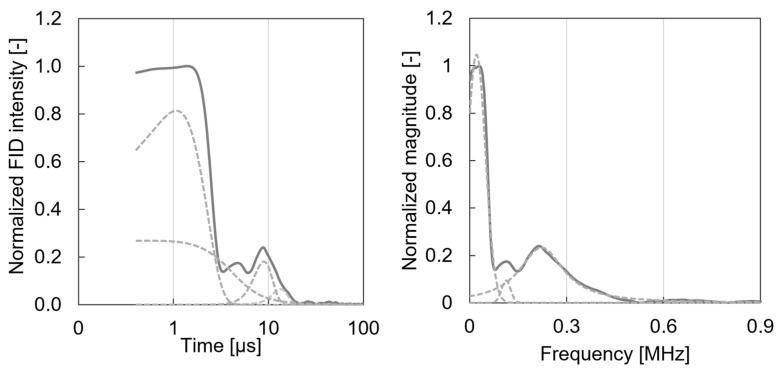
Free induction decay spin-spin relaxation curves for bacterial cellulose (**left** panel) and its Fourier decomposition (**right** panel). The dashed lines represent deconvoluted spectra.

Solid-state low-field proton nuclear magnetic resonance (^1^H-NMR) spectroscopy was employed to investigate the homonuclear spin-spin interactions between hydrogen nuclei within bacterial cellulose molecules, particularly at the carbon 1–2 and 4–5 positions. When diamagnetic BC was subjected to a low-field (0.5 T) external magnetic field and activated by a radio frequency pulse, the resultant free induction decay signal provided insights into proton dynamics. The FID signal for the BC samples could be effectively divided into two primary components: 0.1–3 μs and 3–20 μs (Figure 2). FID relaxation curves for BC were meticulously recorded and subsequently deconvoluted using Voigt-type peaks, which robustly model the cellulose-associated spectral features. The Voigt profile, a convolution of Lorentzian and Gaussian functions, permitted an accurate representation of peaks exhibiting both sharp and broad characteristics. This rigorous deconvolution approach yielded a high R^2^ value of 0.98, indicating a strong correlation between the experimental data and the fitted model.

The FID of the BC sample revealed four distinct deconvoluted peaks. Two prominent peaks, centred at 0.55 and 1.07 μs, were characteristic of highly ordered intracrystalline regions within the microfibrils, representing well-ordered cellulose chain regions with strong inter- and intramolecular hydrogen-bond networks, respectively [16]. Protons exhibiting faster resonance contributed to a slower FID signal decay in the less-ordered cellulose regions centred at 8.9 μs. Additionally, a broad, low intensity spectral line at approximately 30 μs originated from residual bound water. The broader components of the FID signal, represented by wide peaks at later times, yielded valuable insights into the molecular environment of the sample, particularly regarding molecular mobility, viscosity, and structural heterogeneity [17,18,19]. Specifically, variations in relaxation times and spectral linewidths indicated differing degrees of molecular mobility and structural uniformity within the BC sample; higher mobility correlated with longer relaxation times and narrower spectral lines, whilst restricted mobility led to shorter relaxation times and broader lines.

Furthermore, Figure 2 (right panel) presents the NMR Fourier spectrum of the bacterial cellulose sample. To precisely characterise the spectral components, the NMR spectra were further deconvoluted into harmonic Voigt-type signals, as depicted in the figure. This deconvolution process also demonstrated excellent fit, with a high R^2^ value of 0.99, confirming a strong correlation between the experimental data and the fitted model. The spectrum clearly exhibits a broad shoulder at approximately 0.22 MHz and distinct sharp peak at 0.02 MHz. The ratio of the broad (0.053) to sharp (0.057) Voigt peaks area was 93%, correlating with the crystallinity of BC as assessed by proton NMR.

### 3.3. Photoacoustic Characterisation

The analysis conducted using photoacoustic spectroscopy elucidated significant characteristics of the bacterial cellulose samples, offering valuable insights into their optical absorption, thermal properties, and molecular structure. This non-destructive technique facilitated the examination of cellulose in its native state, providing a unique perspective on its complex hierarchical structure.

**Figure 3:**
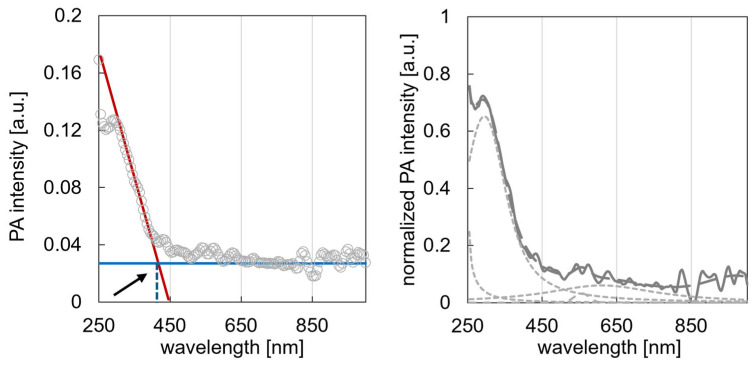
Photoacoustic spectrum of bacterial cellulose. The left panel illustrates the absorption edge at 417 nm, determined by the intersection of derivative spectroscopy (red line) and linear extrapolation (blue line). The right panel displays the deconvoluted spectra.

PAS analysis revealed distinct features corresponding to crystalline and amorphous regions within bacterial cellulose nanofibrils. This approach also facilitated the identification of hydrogen bonding networks and interactions with water molecules, crucial for understanding the structure and properties of cellulose. Scanning across different wavelengths produced absorption spectra that highlighted the characteristic vibrational modes of cellulose molecules as depicted on Figure 3. This spectral analysis not only enabled the identification of specific cellulose polymorphs but also provided insights into the degree of crystallinity of the samples. Such information is particularly valuable when complemented by findings from other analytical methods, contributing to a more comprehensive understanding of cellulose structure and properties. Photoacoustic spectroscopy provides valuable insights into the structural order of bacterial cellulose. Analysis of the PAS spectrum revealed two prominent features: a sharp peak observed at 295 nm, exhibiting a normalised peak height of 0.65 and an area of 87.69 a.u., and a broader peak centred at 610 nm, with a normalised peak height of 0.059 and an area of 24.09 a.u. (Figure 3, right panel). Based on these observations, the sharp peak at 295 nm is associated with the crystalline regions of bacterial cellulose, while the broader peak at 610.31 nm is attributed to the amorphous content. Utilising a peak height method CrI [%] = 100 × (I_cr_ − I_am_)/I_cr_, these distinct spectral characteristics allowed for the calculation of a crystallinity index of 90.8% for the bacterial cellulose sample.

A significant finding from the photoacoustic spectrum analysis was the pronounced increase in signal intensity at a specific wavelength, indicating the material’s absorption edge. Through meticulous examination and data analysis techniques, such as derivative spectroscopy and linear extrapolation, the onset of significant light absorption was determined to be 417 nm. This absorption edge value, representing the mean of these measurements, provides a reliable characterisation of the material’s optical properties. The determination of the absorption edge of cellulose at 417 nm offers crucial insights into its electronic structure and optical properties. This relatively high absorption edge, corresponding to a bandgap of approximately 2.97 eV, indicates stronger insulating properties than those of typical conductors and many other insulators. These unique optical and electronic characteristics distinguish cellulose from materials with lower absorption edges and have important implications for its potential applications in optoelectronic devices and photocatalytic processes.

### 3.4. RDB-PAS Characterisation

**Figure 4:**
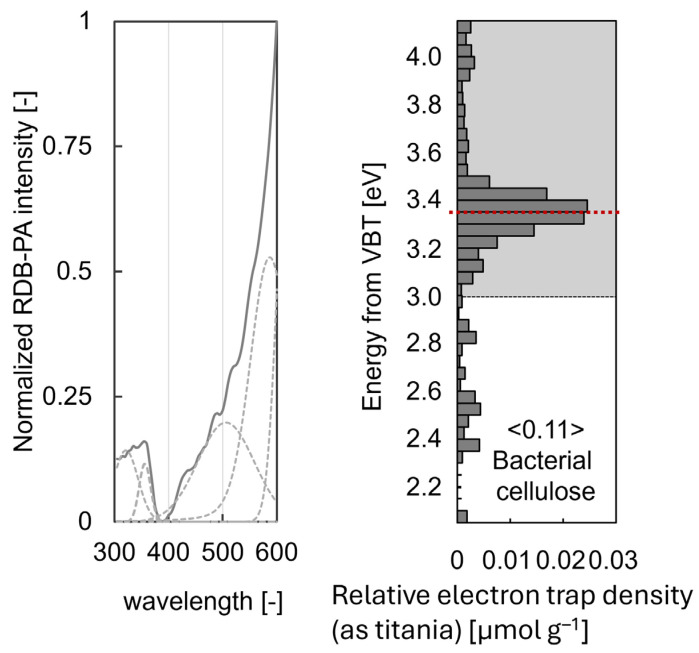
The RDB-PA spectrum and electron trap density of bacterial cellulose; the left panel illustrates the RDB-PA spectrum (continuous line) with deconvoluted spectra (dashed line), and the right panel depicts the electron trap distribution, which reveals defect states within the 3.6–2.9 eV range, featuring a prominent peak at 3.35 eV indicated with the dotted red line.

The band gap of cellulose, which represents the energy difference between its valence and conduction bands, is typically large, ranging from 4.0 to 6.2 eV for pristine cellulose [12,20]. This wide band gap classifies cellulose as an insulator, accounting for its poor electrical conductivity. While the valence band of cellulose, particularly for the Iα type found in bacterial cellulose, is situated between 5.0 and 5.7 eV below the vacuum level, the conduction band, where electrons can move freely, is positioned approximately 3.1 eV above the valence band. However, the presence of defects or structural modifications can significantly alter these energy levels. The experimentally observed defect states in bacterial cellulose, measured using Reversed Double-Beam Photoacoustic Spectroscopy, were found to be in the range of 3.6–2.9 eV as depicted in the right panel of Figure 4. The prominent defect peak observed at 3.35 eV corresponds to a conduction band energy of approximately 2.9 eV, suggesting that the introduction of defects, potentially arising from oxygen vacancies, hydroxyl modifications, or UV-induced structural rearrangements, plays a significant role in altering the electronic structure of bacterial cellulose. These findings indicate that surface dipole effects and hydrogen bonding influence the observed electronic states, leading to defect-mediated modifications of the conduction band position. The strong correlation between defect-induced trap states and the modified conduction band energy provides valuable insights into the optical and electronic properties of bacterial cellulose, particularly under UV irradiation.

### 3.5. XRD Characterisation

**Figure 5:**
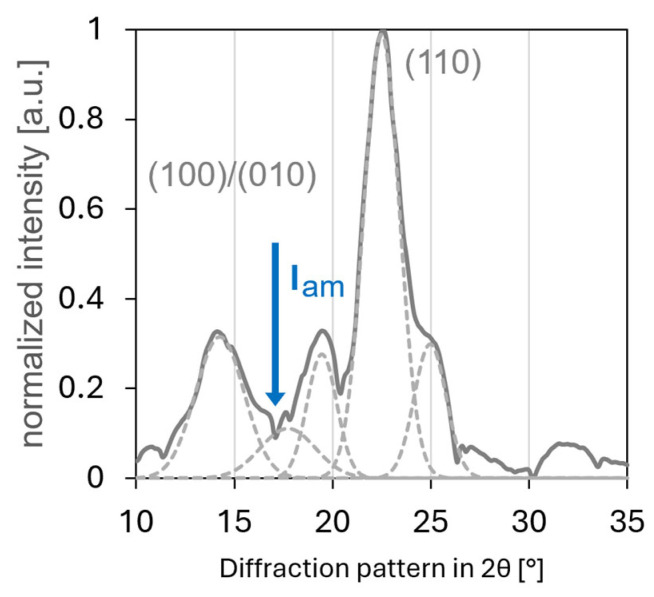
X-ray diffraction spectrum of bacterial cellulose after subtracted the background. The spectrum displays characteristic peaks corresponding to cellulose Iα, including the prominent overlapping (100)/(010) Miller indices and its reflections centred at 19.2° 2θ, and the peak at 22.3° 2θ corresponding the (110) plane. The minimum intensity at 17.08° 2θ represents the amorphous content. The spectrum is also shown with deconvoluted Gaussian curves (dashed lines), used for peak fitting and crystallinity index calculations.

X-ray diffraction analysis revealed the distinctive crystalline characteristics of the bacterial cellulose nanofibrils. The amorphous content in the bacterial cellulose nanofibrils is represented by the minimum intensity at 17.08° 2θ between the overlapped (100) and (010) peaks and the (110) peak in the XRD spectrum [21] (Figure 5).

The overlap of the (100)/(010) peaks in the XRD spectrum of bacterial cellulose can be attributed to several factors related to its crystalline structure. Bacterial cellulose primarily consists of cellulose Iα, which has a triclinic unit cell. The close proximity of the d-spacings corresponding to these planes results in their diffraction peaks appearing at very similar 2θ angles. Additionally, the nanofibrillar nature of bacterial cellulose, with fibrils measuring 10–75 nm in diameter (as observed in the SEM images), can lead to peak broadening due to size effects. This broadening further contributes to the merging of these closely spaced peaks. Although the high degree of crystallinity in bacterial cellulose generally results in sharp peaks, some peak overlaps can occur when crystal planes are similarly oriented. The predominance of the Iα allomorph in bacterial cellulose, as opposed to plant cellulose, may further accentuate this effect owing to its specific crystal structure.

The prominent peak at 19.2° 2θ in the XRD pattern of bacterial cellulose is particularly significant due to the overlap of the (100)/(010) reflections. This overlap is characteristic of the cellulose Iα crystal structure, which predominates in BC. The merging of these peaks indicates the high crystallinity and specific orientation of the cellulose chains within the BC nanofibrils. The intensity of this peak, relative to that of other peaks in the pattern, provides information about the degree of crystallinity and preferential orientation of cellulose molecules in the sample. Furthermore, the width of this peak offers insights into the size of the crystalline domains within the BC structure. The presence and characteristics of this peak are crucial for distinguishing BC from other forms of cellulose and for assessing its structural properties.

These specific crystalline characteristics and observed peak overlaps are influenced by various factors, including the carbon sources and culture media used during the production of bacterial cellulose, which can significantly affect its structural properties [22].

The crystallinity index for the bacterial cellulose was determined using two methods. Based on the equation proposed by Segal et al. [23], the CrI was calculated to be 93.6%, which is in close agreement with the value assessed by proton NMR. For a more detailed analysis, the XRD spectrum was also deconvoluted using Gaussian curves, yielding a high R^2^ fitting value of 0.99. From this deconvolution, the ratio of the areas for the overlapped (100)/(010) peaks at 14.5°, its symmetric counterpart at 19.2°, and the peak at 22.3° against the amorphous content (represented by the minimum intensity at 17.08°) resulted in a crystallinity index of approximately 90%.

## 4. Discussion

The multidisciplinary investigation of bacterial cellulose using a suite of advanced techniques—including scanning electron microscopy, X-ray diffraction, proton nuclear magnetic resonance (^1^H-NMR), photoacoustic spectroscopy, and reversed double-beam photoacoustic spectroscopy—provides a profound insight into its structural, electronic, and phonon dynamic properties.

Initially, the morphological characteristics revealed by SEM highlight the distinctive nanofibrillar network of BC [24], comprising densely interwoven fibrils ranging from 10 to 75 nm in diameter. This intricate, high surface area architecture, along with the observed ribbon-like structures and interconnections between individual fibrils, is fundamental to BC’s properties [25], influencing light-matter interactions and subsequent energy conversion processes. Complementing this, XRD analysis confirmed the highly crystalline nature of BC, identifying characteristic peaks associated with the cellulose Iα allomorph [26]. The observed overlap of the (100)/(010) at 14.5° 2θ and reflections at 19.2° 2θ and the prominent peak at 22.3° 2θ underscore the unique triclinic unit cell structure and the high degree of molecular ordering within the BC nanofibrils [27]. Such structural precision, coupled with the nanofibrillar dimensions and crystalline domain sizes inferred from peak widths, impacts the material’s bulk properties [28] and its interaction with incident photons.

Furthering our understanding of BC’s internal structure, ^1^H-NMR relaxation analysis provided critical insights into the differentiation between ordered and disordered cellulose regions. The distinct peaks at 0.55 and 1.07 μs are indicative of highly ordered intracrystalline domains, reflecting robust inter- and intramolecular hydrogen-bond networks. Conversely, the broader components of the FID signal revealed crucial information concerning molecular mobility, viscosity, and structural heterogeneity within the less ordered regions. These variations in molecular packing and hydrogen bonding directly influence the material’s vibrational modes and pathways for energy dissipation, thereby having significant implications for photo-conversion efficiency. The presence of residual bound water, indicated by a spectral line at approximately 30 μs, also suggests an additional factor influencing molecular dynamics.

The optoelectronic behaviour and defect dynamics of BC were comprehensively explored through PAS and RDB-PAS. PAS confirmed BC’s insulating nature by revealing an optical bandgap of 2.97 eV, corresponding to an absorption edge at 417 nm. Further analysis of the PAS spectrum allowed for differentiation between crystalline and amorphous regions. A sharp peak at 295 nm was associated with the crystalline domains of bacterial cellulose, while a broader peak centred at 610 nm was attributed to its amorphous content. Utilising a peak height method, these distinct spectral characteristics enabled the calculation of a crystallinity index of 90.8% from the PAS data.

These variations observed in photoacoustic measurements stem from the presence of crystalline and amorphous regions within the bacterial cellulose, which influence light absorption and heat conversion processes. Additionally, defect states identified in the range of 3.6–2.9 eV through RDB-PAS spectroscopy, with a prominent peak at 3.35 eV, can affect optical absorption properties and phonon generation processes. The high surface area of bacterial cellulose nanofibrils enhances surface-related phenomena, such as surface dipole effects and hydrogen bonding, which may differentially affect photon-to-phonon conversion processes in PAS and RDB-PAS measurements. Furthermore, the RDB-PAS technique can be used to investigate photoinduced changes in absorption, revealing the dynamic processes that occur upon light exposure, such as trap filling and electron–hole pair dynamics. The differing sensitivities of PAS and RDB-PAS spectroscopy to various energy levels provide complementary insights into the electronic structure of bacterial cellulose, with PAS spectroscopy being sensitive to the overall absorption profile and RDB-PA selectively probing specific energy levels associated with trap states. Collectively, these factors contribute to the observed differences between the PAS and RDB-PAS spectra, offering comprehensive insights into the structural and electronic properties of bacterial cellulose.

Crucially, our interpretation of these defect states and their role in the observed modification of the optical bandgap is firmly rooted in the direct experimental evidence provided by the RDB-PAS measurements. This technique uniquely probes these trap states and their photoinduced dynamics, ensuring a strong, empirically driven link between our direct observations and their mechanistic interpretation within the electronic structure of bacterial cellulose.

The interplay between these structural, molecular, and electronic characteristics is pivotal to understanding the mechanisms of photon-to-phonon conversion in bacterial cellulose. Incident photons absorbed by BC can excite electrons, leading to the creation of electron–hole pairs. While radiative recombination is an ideal outcome, non-radiative pathways, such as phonon-assisted processes, are significantly influenced by the material’s inherent properties. The crystalline and amorphous regions, as identified by XRD and NMR, dictate the efficiency of light absorption and subsequent heat conversion processes. Furthermore, the identified defect states act as crucial intermediaries, facilitating energy transfer from excited electrons to lattice vibrations (phonons). RDB-PAS, in particular, offers the capability to investigate photoinduced changes in absorption, thereby revealing the dynamic processes of trap filling and electron–hole pair recombination which are central to understanding non-radiative energy dissipation.

It is important to acknowledge that non-radiative recombination is a complex phenomenon involving a diverse array of mechanisms beyond those explicitly detailed, such as Auger recombination or phonon-assisted processes. While our study focuses on the collective outcome and the mechanisms most pertinent to the observed photothermal phenomena in BC, other non-radiative pathways undoubtedly contribute to the overall energy dissipation. Our techniques primarily probe the sum effect leading to thermal energy generation, and future studies could delve deeper into distinguishing and quantifying the contributions of various individual non-radiative pathways.

Collectively, this integrated approach not only characterises BC’s fundamental properties but also provides a robust framework for comprehending how its unique nanofibrillar structure, molecular ordering, and electronic defect landscape govern the conversion of photonic energy into phonons. This foundational understanding is vital for tailoring BC’s properties for advanced applications in optoelectronics and energy harvesting.

## 5. Conclusions

The comprehensive analysis of bacterial cellulose using multiple spectroscopic and diffraction techniques has provided valuable insights into its structural, electronic, and morphological properties. Photoacoustic spectroscopy revealed an optical bandgap of 2.97 eV, confirming the insulating nature of BC. Reversed double-beam photoacoustic spectroscopy identified defect states between 3.6 and 2.9 eV, with a prominent peak at 3.35 eV, likely attributed to oxygen vacancies, hydroxyl modifications, or UV-induced rearrangements.

Furthermore, ^1^H-NMR relaxation analysis successfully differentiated between ordered and disordered cellulose regions in BC, with peaks at 0.55 and 1.07 μs indicating highly ordered intracrystalline regions. The broader components provided crucial information regarding molecular mobility and structural heterogeneity. Concurrently, X-ray diffraction confirmed the crystalline nature of BC, identifying characteristic peaks associated with cellulose Iα, particularly the overlap of (1–10) and reflections at 14.5° 2θ.

The integration of these complementary techniques offers a truly multidimensional understanding of BC’s properties. PAS and RDB-PAS elucidate optical absorption and defect states, ^1^H-NMR reveals molecular ordering, and XRD confirms the crystalline structure. This holistic approach provides a robust foundation for future research and potential applications of bacterial cellulose across diverse fields, including materials science, bioengineering, and nanotechnology. Future studies could therefore concentrate on targeted defect engineering, building upon the identified defect states, to fine-tune the bandgap and enhance photo-conversion efficiency for specific optoelectronic or bioelectronic applications.

## Data Availability

The original contributions of this study are included in the article. Further inquiries can be directed to the corresponding author.
